# A_2A_R inhibition in alleviating spatial recognition memory impairment after TBI is associated with improvement in autophagic flux in RSC

**DOI:** 10.1111/jcmm.15361

**Published:** 2020-05-12

**Authors:** Xu‐Jia Zeng, Ping Li, Ya‐Lei Ning, Yan Zhao, Yan Peng, Nan Yang, Ya‐Wei Xu, Jiang‐Fan Chen, Yuan‐Guo Zhou

**Affiliations:** ^1^ State Key Laboratory of Trauma, Burn, and Combined Injury Department of Occupational Disease Daping Hospital Army Medical University Chongqing China; ^2^ Department of Neurology and Pharmacology Boston University School of Medicine Boston MA USA

**Keywords:** lysosomal biogenesis, manganese‐enhanced magnetic resonance imaging, neuronal apoptosis, retrosplenial cortex, transcription factor EB

## Abstract

Spatial recognition memory impairment is an important complication after traumatic brain injury (TBI). We previously found that spatial recognition memory impairment can be alleviated in adenosine A_2A_ receptor knockout (A_2A_R KO) mice after TBI, but the mechanism remains unclear. In the current study, we used manganese‐enhanced magnetic resonance imaging and the Y‐maze test to determine whether the electrical activity of neurons in the retrosplenial cortex (RSC) was reduced and spatial recognition memory was impaired in wild‐type (WT) mice after moderate TBI. Furthermore, spatial recognition memory was damaged by optogenetically inhibiting the electrical activity of RSC neurons in WT mice. Additionally, the electrical activity of RSC neurons was significantly increased and spatial recognition memory impairment was reduced in A_2A_R KO mice after moderate TBI. Specific inhibition of A_2A_R in the ipsilateral RSC alleviated the impairment in spatial recognition memory in WT mice. In addition, A_2A_R KO improved autophagic flux in the ipsilateral RSC after injury. In primary cultured neurons, activation of A_2A_R reduced lysosomal‐associated membrane protein 1 and cathepsin D (CTSD) levels, increased phosphorylated protein kinase A and phosphorylated extracellular signal‐regulated kinase 2 levels, reduced transcription factor EB (TFEB) nuclear localization and impaired autophagic flux. These results suggest that the impairment of spatial recognition memory after TBI may be associated with impaired autophagic flux in the RSC and that A_2A_R activation may reduce lysosomal biogenesis through the PKA/ERK2/TFEB pathway to impair autophagic flux.

## 1 INTRODUCTION

Spatial recognition memory is a type of spatial memory that allows animals to recognize a new environment.^1^, ^2^ Traumatic brain injury (TBI) is one of the most common brain injuries; the impaired spatial memory caused by TBI severely reduces the survival of patients and poses a significant burden to their families and society.^3^, ^4^, ^5^ Although a variety of methods have been used to alleviate this impairment, few effective and feasible treatments are available.^4^, ^6^, ^7^ The adenosine A_2A_ receptor (A_2A_R) is a type of G protein‐coupled receptor that can be activated by markedly increased adenosine after TBI. Inhibition of A_2A_R has a protective effect on both acute brain injury and chronic cognitive impairment, including spatial recognition memory impairment after TBI.^8^, ^9^, ^10^ However, the mechanism of A_2A_R inhibition in improving spatial recognition memory impairment is still unclear. Cell death has been reported as an important cause of spatial recognition memory impairment.^4^, ^6^, ^7^ Additionally, we previously found that improving autophagic flux is an important factor for reducing cell death in A_2A_R knockout (KO) mice after moderate TBI, which provides new support for the neuroprotective effects of A_2A_R.^11^ Thus, we hypothesize that inhibiting A_2A_R activation to improve autophagic flux may be associated with improving spatial recognition memory impairment after TBI.

To answer these questions, we first used manganese‐enhanced magnetic resonance imaging (MEMRI) to clearly identify the retrosplenial cortex (RSC) as the significantly changed region in wild‐type (WT) mice after moderate TBI while A_2A_R KO mice could reverse this change. Second, we determined that the decreased function of the RSC is important for the impairment of spatial recognition memory by optogenetically inhibiting the electrical activity of retrosplenial cortical neurons in WT mice. Next, we detected autophagic flux in the RSC of WT and A_2A_R KO mice after TBI and found that impaired autophagic flux in the RSC induced by A_2A_R activation resulted in spatial recognition memory impairment. Finally, we explored the mechanism of impaired autophagic flux induced by A_2A_R in vivo and in vitro.

## 2 METHODS AND MATERIALS

### 2.1 Animals

The A_2A_R KO mice used in this study were provided by Dr Chen.^12^, ^13^ Male mice aged 8‐12 weeks and weighing 22‐26 g were used in our experiments. The mice were allocated to the sham, mild TBI and moderate TBI groups using a random number table. All animal procedures were reviewed and approved by the Administration of Affairs Concerning Experimental Animals Guidelines of The Third Military Medical University.

### 2.2 TBI model

The controlled cortical impact method was used to produce mild and moderate TBI models according to the methods described in our previous protocol.^11^

### 2.3 Primary culture of cortical neurons

Mouse cortical neuron cultures were prepared as previously described.^14^ Briefly, embryos were obtained from mice at 18 days of gestation anaesthetized under pentobarbital sodium. The cortex was isolated using sterile microforceps under a stereomicroscope and treated with 0.25% trypsin for 15 minutes at 37°C. Trypsinization was stopped by adding 10% FBS, and the cell suspensions were seeded at 1 × 10^5^ cells cm^−2^ in neurobasal medium (Invitrogen) containing 2% B27 supplement (Invitrogen), 0.5 mmol/L l‐glutamine and 25 μmol/L l‐glutamic acid. Half of the medium was replaced with B27/neurobasal without l‐glutamic acid 4 days later. The neurons were used after 14 days of culture.

### 2.4 Y‐maze test

Animals were tested for spatial recognition memory in a Y‐maze as previously described.^15^ Briefly, the three identical arms of a Y‐maze were randomly designated as follows: the start arm (always open), in which the mouse started to explore; the novel arm, which was blocked during the 1st trial but opens during the 2nd trial; and the other arm (always open). The test consisted of two trials separated by an intertrial interval (2 hours). In the first training (acquisition) trial, mice were placed in the maze facing the end of a pseudorandomly chosen start arm and allowed to explore the maze for 5 minutes with the novel arm closed. Mice were then returned to their home cage until the second (retrieval) trial, during which they could freely explore all three arms of the maze. The time spent in each arm and the number of entries were measured and analysed from video recordings (Ethovision, Noldus Information Technology Inc, Leesburg, VA, USA). Mice were required to enter an arm with all four paws for it to be counted as an entry. Entering the novel arm more frequently and exploring it for longer periods of time indicated intact spatial recognition memory. In our study, spatial recognition memory was tested on the 7th day after TBI.

### 2.5 MnCl_2_ administration

Continuous administration was achieved via ALZET mini‐osmotic pumps (model 1007D; DURECT Corporation, Cupertino, CA, USA). The osmotic pumps were implanted into the abdominal cavity of WT and A_2A_R KO mice immediately after moderate TBI. Over 7 days, this resulted in cumulative doses of 400 mg/kg MnCl_2_ in each mouse. Then, the mini‐osmotic pumps were removed on the 7th day for MRI compatibility.

### 2.6 MRI data acquisition

MRI was performed on the 7th day following TBI and MnCl_2_ administration using a 7‐T MRI scanner (BioSpec 70/20 USR, Bruker, Germany). After anaesthesia with 1.5% pentobarbital sodium, axial T1‐weighted images (repetition time  = 350 ms, echo time  = 11 ms, slice thickness = 1.0 mm and field of view [FOV]  = 4.0 × 4.0 cm) were acquired. Three MRI images were selected for each mouse and the region of interest in each image was a 0.8 × 0.8 mm area selected from the ipsilateral RSC.

### 2.7 Immunofluorescence, immunohistochemistry and TUNEL assays

Immunofluorescence, immunohistochemistry and TUNEL assays were performed according to previously described methods.^11^ Briefly, sections (WT + sham, KO + sham, WT + TBI and KO + TBI groups, n = 3) or cultured cortical neurons (control, CGS21680, CGS21680 + H89 and CGS21680 + PD98059 groups, n = 3) from each group were incubated with the following primary antibodies overnight at 4°C: anti‐LC3 (1:200, Abcam, ab64781), anti‐caspase 3 (1:100, Santa Cruz Biotechnology, sc‐271759), anti‐cFos (1:200, Abcam, ab190289), anti‐NeuN (1:200, Abcam, ab104224), anti‐A_2A_R (1:200, Abcam, ab3461), anti‐SQSTM1 (1:200; Abcam, ab91526) and anti‐transcription factor EB (TFEB) (1:50, Santa Cruz Biotechnology, sc‐48784). For immunofluorescence analyses, the sections were rewarmed for 30 minutes, washed with PBS and then incubated with Alexa Fluor 488‐ or Cy3‐conjugated secondary antibodies (1:200; Abcam) for 1 hour at room temperature. The sections were then washed and mounted on slides using UltraCruz™ hard‐set mounting medium with DAPI (Santa Cruz Biotechnology, sc‐359850). For immunohistochemical analyses, after the sections were incubated with secondary antibodies, a streptavidin/peroxidase and diaminobenzidine substrate kit (ZSGB‐BIO, Beijing, China) was used to visualize the results. TUNEL assays were performed on paraffin‐embedded brain sections using an In Situ Cell Death Detection Kit, TMR red (Roche, 12156792910, California, USA). The total number of brain cells and the number of TUNEL‐positive cells were manually counted; the apoptotic index was defined as the percentage of TUNEL‐positive cells relative to the total number of brain cells.^16^, ^17^ We used previously described methods and Image‐Pro Plus 6.0 software (Media Cybernetics, Rockville, MD, USA) to analyse the results.^18^, ^19^ All measurements were obtained from one field from each of three slices per mouse.

### 2.8 Western blot assays

Western blot assays were performed according to previously described methods.^11^ The membranes were probed with the following primary antibodies overnight at 4°C: anti‐cathepsin D (CTSD) (1:2000, Abcam, ab75852), anti‐lysosomal‐associated membrane protein 1 (LAMP1) (1:2000, Abcam, ab24170), anti‐phosphorylated protein kinase A (p‐PKA) (1:5000, Abcam, ab32390), anti‐PKA (1:2000, Abcam, ab75993), anti‐phosphorylated extracellular signal‐regulated kinase 2 (p‐ERK2) (1:1000, Abcam, ab201015), anti‐ERK2 (1:1000, Abcam, ab32081), anti‐TFEB (1:1000, Santa Cruz Biotechnology, sc‐48784), anti‐histone 2B (1:2000, Abcam, ab40886), anti‐LC3B (1:1000; Sigma, L7543), anti‐SQSTM1 (1:1000; Abcam, ab91526), anti‐Beclin1 (1:1000, Santa Cruz Biotechnology, sc‐11427), anti‐Atg5 (1:1000; Abcam, ab78073), anti‐phosphorylated TFEB (Ser142) (1:1000; Merck, ABE1971‐l), anti‐peroxisome proliferator‐activated receptor gamma co‐activator 1‐alpha (PGC‐1α) (1:1000; Abcam, ab54481) and anti‐β‐actin (1:5000, Abcam, ab8226). After incubation with horseradish peroxidase‐conjugated secondary antibodies, the membranes were visualized using SuperSignal Chemiluminescent Substrates (Thermo Fisher Scientific, 34080, Massachusetts, USA).

### 2.9 Isolation of nuclear and cytoplasmic extracts

Nuclear‐cytoplasmic fractionation was conducted using the NE‐PER Nuclear and Cytoplasmic Extraction Reagents Kit (Thermo Fisher Scientific, 78833) according to the manufacturer's protocol.

### 2.10 Real‐time PCR

Total RNA was isolated from the ipsilateral RSC and cultured cortical neurons from each group using TRIzol (Invitrogen, 10296010) and then reverse‐transcribed. The SYBR Green Kit (TaKaRa Bio Inc, RR820L) was used for quantitative PCR. The following primers were used to measure mRNA levels: *lamp1* (forward: 5′‐AGTGGCCC‐TAAGAACATGACC‐3′ and reverse: 5′‐AGTGTATGTCCTCTTCCAAAAGC‐3′) and *ctsd* (forward: 5′‐CCACTGTCAGGGAACTGGAT‐3′ and reverse: 5′‐CTCCTTCAGACA‐GGCAGAGG‐3′).

### 2.11 Chloroquine injection

This experiment was performed according to our previously described methods.^11^

### 2.12 ZM241385 injection

The injection system was described in our previous article.^11^ The A_2A_R antagonist ZM241385 (1 mg/kg) was administered into the ipsilateral RSC immediately after moderate TBI was induced.

### 2.13 Autophagic flux measurement

Adeno‐associated virus (AAV)‐red fluorescent protein (RFP)‐green fluorescent protein (GFP)‐LC3 and adenovirus (Ad)‐RFP‐GFP‐LC3 were purchased from Hanbio (Shanghai, China). AAV‐RFP‐GFP‐LC3 was stereotaxically injected into the ipsilateral RSC of mice (2 μL) 3 weeks before TBI. Then, the brains were cut into coronal cryosections (30 μm). Ad‐RFP‐GFP‐LC3 was added to the wells containing primary cultured neurons for 3 days before drug administration. Finally, the sections and cultured neurons were photographed, and the relative fluorescence intensity was analysed with Image‐Pro Plus 6.0 software (Media Cybernetics, Rockville, MD, USA). GFP, but not RFP, is degraded in an acidic environment. Thus, yellow spots (merge of red and green fluorescence) indicate autophagosomes, whereas red spots indicate autolysosomes. If autophagy is activated and the autophagic flux is normal, the red signal will dominate over the yellow signal. However, if autophagic flux is impaired, more yellow signal than red signal will be observed.

### 2.14 Optogenetic inhibition experiment

Adeno‐associated virus‐CaMKIIα‐Archaerhodopsin (Arch)‐yellow fluorescent protein (EYFP) and AAV‐CaMKIIα‐EYFP were purchased from OBiO (Shanghai, China); AAV‐CaMKIIα‐Arch‐EYFP and AAV‐CaMKIIα‐EYFP were stereotaxically injected into the ipsilateral cortex of mice (2 μL) 8 weeks before the experiments. Then, we implanted optical cannulae into the RSC; the mice were habituated to optical cannulae connected to an optical patch cable without laser stimulation for 30 minutes per day for 7 days in the Y‐maze. Next, mice were placed in the Y‐maze with optical cannulae connected to the optical patch cable and a green laser on test day. During the Y‐maze test, which was used to detect spatial recognition memory, green light illumination with a green laser (525 nm, 3.85 mW/mm^2^ at the cannulae tip) was applied for 5 minutes through the optical cannulae to activate Arch in the RSC.

### 2.15 Pharmacological treatments

To elucidate the signalling pathway associated with A_2A_R activation, the A_2A_R agonist CGS21680 (100 nmol/L) was added to primary cultured cortical neurons that were exposed to oxygen‐glucose deprivation for 2 hours.^20^ To explore the roles of PKA and ERK in the A_2A_R‐induced impairment of autophagic flux, the PKA inhibitor H89 (10 μmol/L) or the ERK inhibitor PD98059 (50 μmol/L) was administered in addition to CGS21680 treatment and oxygen‐glucose deprivation for 2 hours. Then, the neurons were collected for subsequent experiments.

### 2.16 CTSD activity assay

The CTSD activity assay was performed by using a kit from Abcam (ab65302). Briefly, mice were anaesthetized, perfused with ice‐cold saline and decapitated, and cortical tissue from the RSC was dissected and homogenized in ice‐cold cell lysis buffer provided in the kit. Tissue homogenates were centrifuged at 15 000 × *g* for 5 minutes at 4°C. The protein concentration was estimated by the BCA method. Fifty micrograms of protein was incubated with the CTSD substrate mixture at 37°C for 1 hour. Fluorescence released from the synthetic substrate by CTSD in the tissue was estimated with a fluorescence plate reader (Thermo Fisher Scientific, Varioskan Flash, Massachusetts, USA) at Ex/Em = 328/460 nm.

### 2.17 Statistical analysis

All results are shown as the mean ± SD. All of the results from this study were analysed by researchers who were blinded to the experimental grouping. For comparisons between two independent samples that conform to the normal distribution and homogeneity of variance, we used Student's *t* tests to analyse the data, while we used the rank sum test for discontinuous variables. Two‐way ANOVA followed by Bonferroni's post hoc test was used for comparisons of more than two samples with two variables. One‐way ANOVA followed by Bonferroni's post hoc test was used for comparisons of more than two independent samples.

## 3 RESULTS

### 3.1 Spatial recognition memory is impaired after TBI, which may be related to impaired RSC function

**FIGURE 1:**
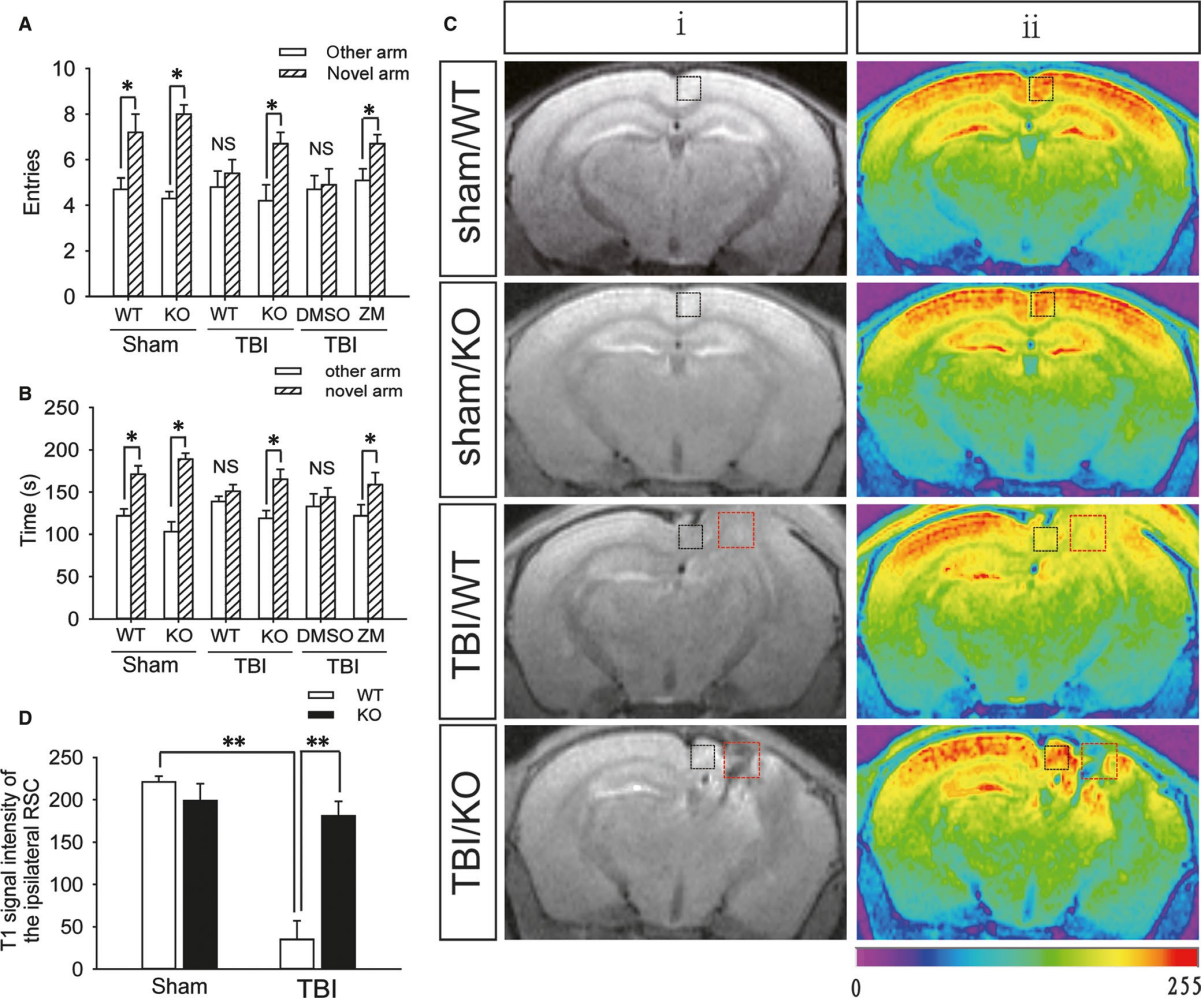
A_2A_R knockout is beneficial to the function of the retrosplenial cortex and spatial recognition memory after TBI. A, The number of entries into the novel arm and other arm by mice in the sham (WT and KO), WT + TBI, KO + TBI, WT + TBI +DMSO and WT + TBI + ZM241385 groups; DMSO: dimethyl sulfoxide and ZM241385: A_2A_R antagonist. Data are presented as the means ± SDs, n = 15, novel arm vs other arm, *P < 0.05. B, Exploration time in the novel arm and other arm by mice from the sham (WT and KO), WT + TBI, KO + TBI, WT + TBI +DMSO and WT + TBI +ZM241385 groups. Data are presented as the means ± SDs, n = 15, novel arm vs other arm, *P < 0.05. C, T1‐weighted MEMRI of the brains of mice from the sham (WT and KO), WT + TBI and KO + TBI groups; the images in (ii) are colour‐coded versions of the images from (i) to better visualize the enhancement patterns. An increase in the red signal represents stronger neuronal activity. The black square frame indicates the ipsilateral retrosplenial cortex and the red square frame indicates the injured part. D, Quantification of the T1 signal intensity in the ipsilateral retrosplenial cortex shown in (C). Data are presented as the means ± SDs, n = 3, WT + TBI group vs KO + TBI group, **P < 0.01. KO, knockout; MEMRI, manganese‐enhanced magnetic resonance imaging; TBI, traumatic brain injury; WT, wild‐type

**FIGURE 2:**
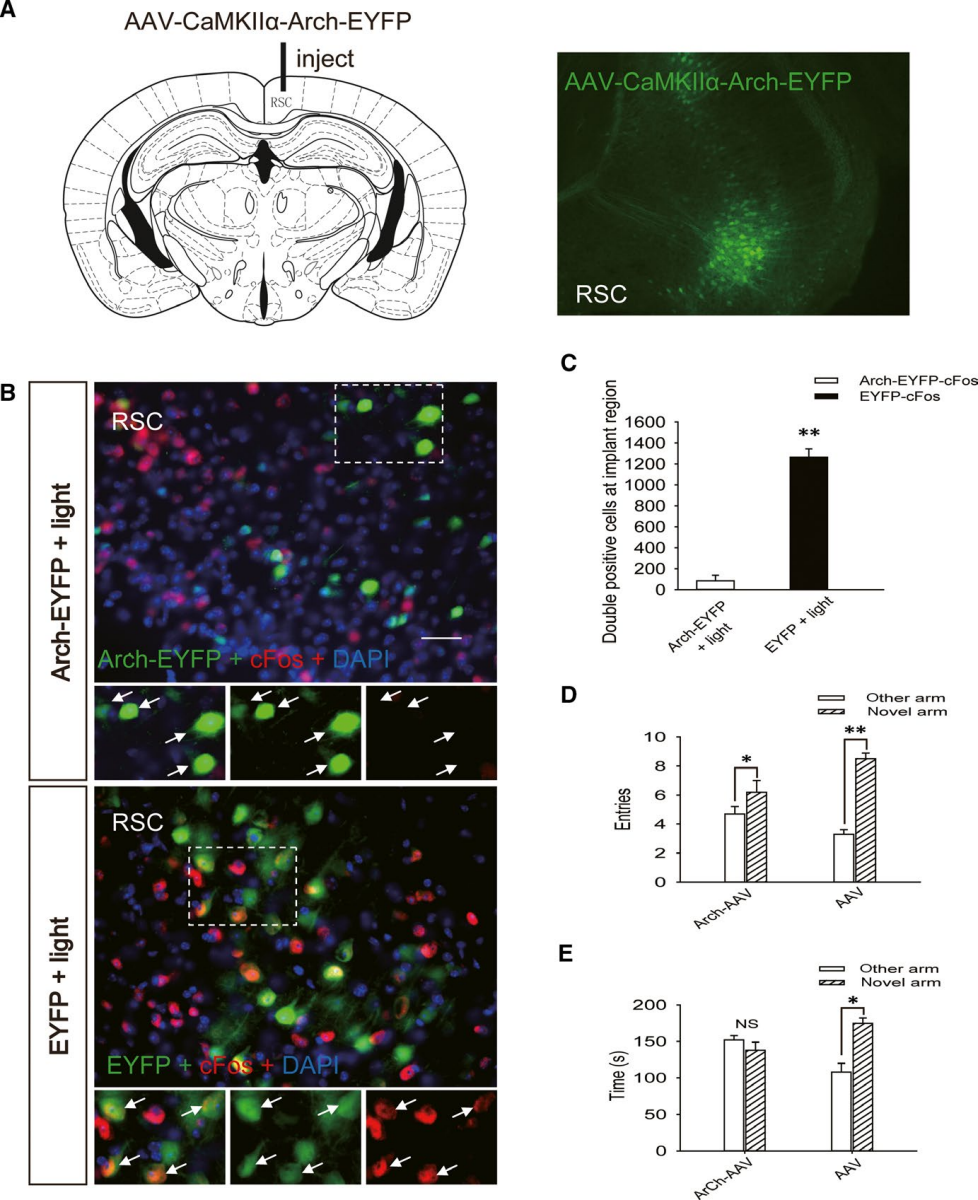
Impairment of spatial recognition memory through inhibition of neuronal activity in the retrosplenial cortex. A, Map of the virus injection site and an image of a retrosplenial cortex section obtained from a WT mouse injected with AAV‐CaMKIIα‐Arch‐EYFP. Green fluorescence represents Arch‐EYFP expression in neurons in the retrosplenial cortex. B, Images of retrosplenial cortex sections obtained from WT mice injected with AAV‐CaMKIIα‐Arch‐EYFP and AAV‐CaMKIIα‐EYFP after application of green light illumination. Sections were stained using an anti‐cFos antibody; green fluorescence represents Arch‐EYFP and red fluorescence represents cFos. Scale bar = 50 μm. C, Quantification of the number of cells that were positive for both Arch‐EYFP and cFos and for both EYFP and cFos in the ipsilateral retrosplenial cortex sections shown in (B). Data are presented as the means ± SDs, n = 3, Arch‐EYFP + light group vs EYFP + light group, **P < 0.01. More than 1000 cells were quantified in each section from each mouse in each experiment. D, The number of entries into the novel arm and the other arm by mice in the AAV‐CaMKIIα‐Arch‐EYFP + light and AAV‐CaMKIIα‐EYFP + light groups. Data are presented as the means ± SDs, n = 9, novel arm vs other arm, *P < 0.05 and **P < 0.01. E, Exploration time in the novel and other arms by the mice in the AAV‐CaMKIIα‐Arch‐EYFP + light and AAV‐CaMKIIα‐EYFP + light groups. Data are presented as the means ± SDs, n = 9, novel arm vs other arm, *P < 0.05. AAV, Adeno‐associated virus; WT, wild‐type

In the spatial recognition memory test, WT mice in the sham group entered the novel arm more often and spent more time in the novel arm than mice in the other groups, while the number of entries into and exploration time in the novel arm were decreased in the group of WT mice with moderate TBI (Figure 1A,B). Due to the recovery of motor function in mice, spatial recognition memory was tested on the 7th day after TBI. The T1 MEMRI signal in the ipsilateral RSC was significantly decreased in the WT mice on the 7th day after TBI compared with that in the WT mice in the sham group (Figure 1C,D). AAV‐CaMKIIα‐Arch‐EYFP was expressed in the RSC of mice for 2 months, and stable expression was confirmed (Figure 2A). The colocalization of Arch‐EYFP and cFos was significantly decreased (Figure 2B,C), and the number of entries into and exploration time in the novel arm test were decreased (Figure 2D,E) after the light‐induced activation of Arch.

### 3.2 KO or inhibition of A_2A_R in the RSC can alleviate spatial recognition memory impairment

**FIGURE 3:**
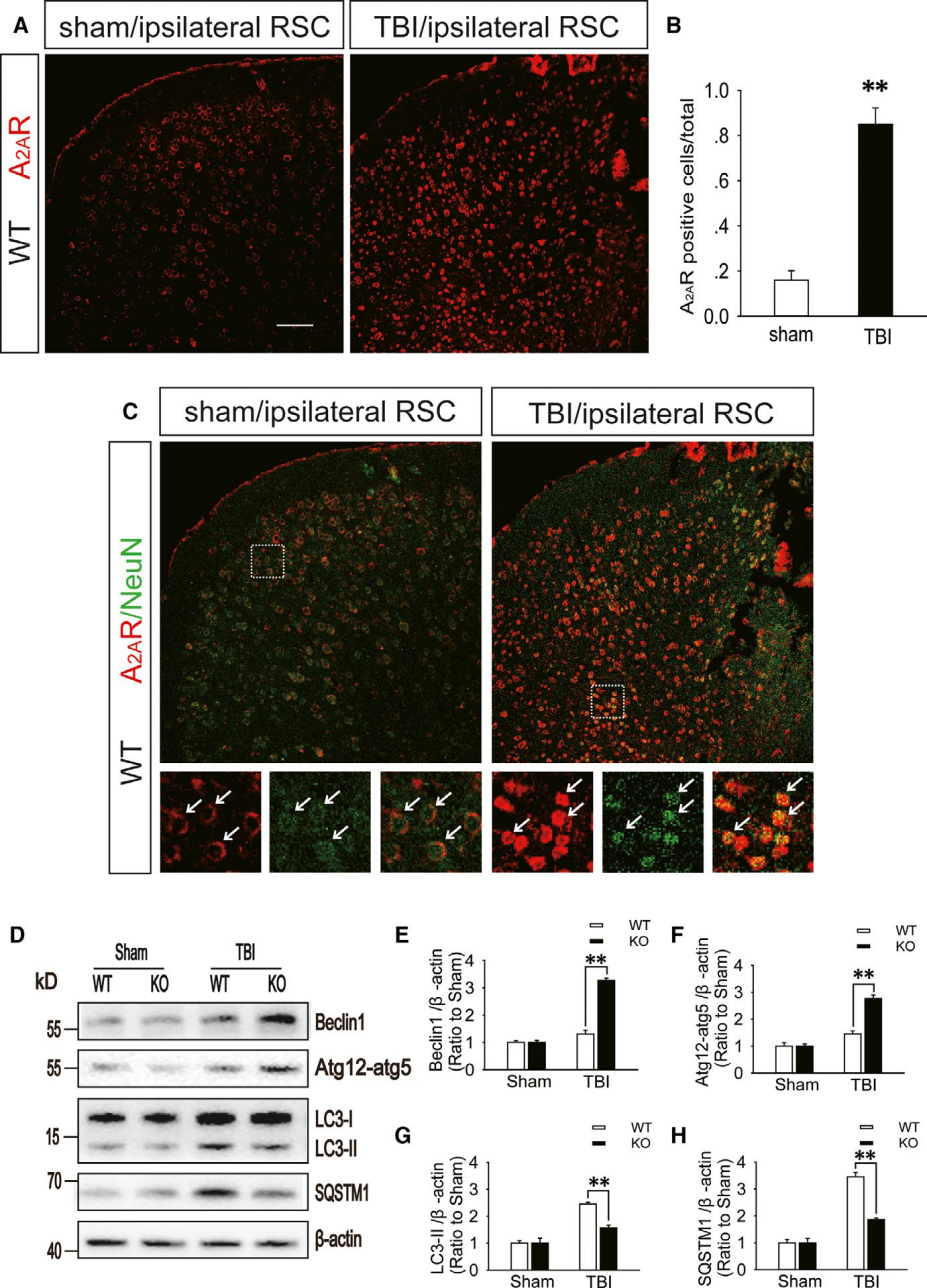
A_2A_R levels are increased in the retrosplenial cortex after TBI. A, Images of ipsilateral retrosplenial cortex sections obtained from WT mice subjected to sham surgery or moderate TBI. The sections were stained using an anti‐A_2A_R antibody; red fluorescence represents A_2A_R. Scale bar = 100 μm. B, Quantification of the number of A_2A_R‐positive cells in the ipsilateral retrosplenial cortex sections shown in (A). Data are presented as the means ± SDs, n = 3, TBI group vs sham group, **P < 0.01. More than 1000 cells were quantified in each section from each mouse in each experiment. C, Images of ipsilateral retrosplenial cortex sections obtained from WT mice subjected to sham surgery or moderate TBI. Sections were stained using an anti‐A_2A_R antibody and an anti‐NeuN antibody; red fluorescence represents A_2A_R and green fluorescence represents NeuN. Arrows indicate the cells presenting both red and green fluorescence. D, Western blot analysis of the protein levels of Beclin1, ATG12‐ATG5 conjugate, LC3 and SQSTM1 in RSC tissue lysates obtained from sham and TBI mice 1 d after injury. E‐H, The Beclin1, ATG12‐ATG5 conjugate, LC3 and SQSTM1 levels shown in (D) were quantified and normalized to β‐actin. Data shown are presented as means ± SDs, n = 5‐6, KO + TBI group vs WT + TBI group, **P < 0.01. KO, knockout; RSC, retrosplenial cortex; TBI, traumatic brain injury; WT, wild‐type

The number of entries into and exploration time in the novel arm were increased in A_2A_R KO mice compared with those in the WT mice after moderate TBI (Figure 1A,B), and the T1 MEMRI signal in the ipsilateral RSC was significantly enhanced in the A_2A_R KO mice (Figure 1C,D) on the 7th day after TBI. Furthermore, the immunofluorescence results showed that compared to that in the sham group, the number of A_2A_R‐positive cells in the RSC of WT mice was significantly increased (Figure 3A,B) and that the A_2A_R levels in retrosplenial cortical neurons were up‐regulated (Figure 3C) on the first day after moderate TBI. Additionally, injection of the A_2A_R antagonist ZM241385 into the ipsilateral RSC of WT mice immediately after moderate TBI significantly increased the number of entries into and exploration time in the novel arm on the 7th day after injury (Figure 1A,B).

### 3.3 Impaired autophagic flux in the RSC may lead to the impairment of spatial recognition memory in WT mice after TBI, which is rescued in A_2A_R KO mice

**FIGURE 4:**
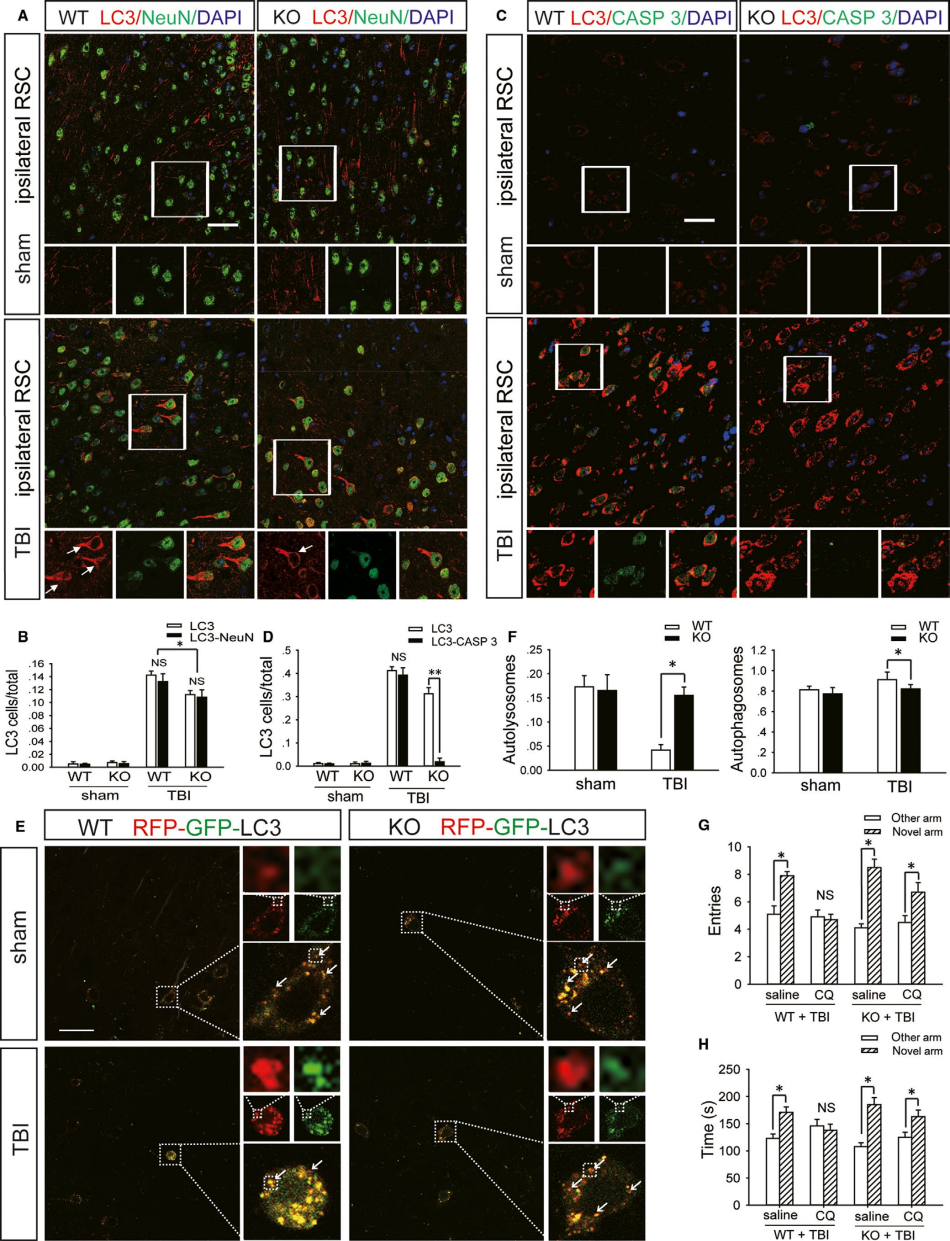
A_2A_R KO can reduce the impairment of spatial recognition memory by improving autophagic flux in retrosplenial cortical neurons after TBI. A, Images of sections of the ipsilateral retrosplenial cortex of WT and KO mice in the sham and moderate TBI groups. The sections were stained with antibodies against LC3 and NeuN; red fluorescence represents LC3 and green fluorescence represents NeuN. Scale bar = 40 μm. B, Quantification of the number of cells that were positive for LC3 only and for both LC3 and NeuN in ipsilateral retrosplenial cortex sections shown in (A). Data are presented as the means ± SDs, n = 3, KO + TBI group vs WT + TBI group, **P < 0.01. More than 1000 cells were quantified in each section from each mouse in each experiment. C, Images of sections of the ipsilateral retrosplenial cortex of WT and KO mice in the sham and moderate TBI groups. Sections were stained with antibodies against LC3 and caspase‐3 (CASP 3); red fluorescence represents LC3 and green fluorescence represents CASP 3. Scale bar = 40 μm. D, Quantification of the number of cells that were positive for LC3 only and for both LC3 and CASP 3 in the ipsilateral retrosplenial cortex sections shown in (C). Data are presented as the means ± SD, n = 3, KO + TBI group vs WT + TBI group, **P < 0.01. More than 1000 cells were quantified in each section from each mouse in each experiment. E, Images of ipsilateral retrosplenial cortex sections obtained from WT and KO mice injected with AAV‐RFP‐GFP‐LC3 in the sham and moderate TBI groups. Arrows indicate the presence of red puncta. Scale bar = 40 μm. F, Quantification of the percentage of autolysosomes (red puncta/total puncta) and autophagosomes (yellow (red + green) puncta/total puncta) in the images shown in (E). Data are presented as the means ± SDs, n = 3, KO + TBI group vs WT + TBI group, *P < 0.05. More than 100 cells were quantified in each section from each mouse in each experiment. G, The number of entries into the novel arm and other arm by WT and KO mice that were subjected to mild TBI and administered CQ or saline; CQ: chloroquine. Data are presented as the means ± SDs, n = 9, novel arm vs other arm, *P < 0.05. H, Exploration time in the novel arm and other arm by WT and KO mice that were subjected to mild TBI and administered CQ or saline. Data are presented as the means ± SDs, n = 9, novel arm vs other arm, *P < 0.05. AAV, Adeno‐associated virus; GFP, green fluorescent protein; KO, knockout; RFP, red fluorescent protein; TBI, traumatic brain injury; WT, wild‐type

Western blot analysis showed that compared with those in the WT mice on day 1 after moderate TBI, the protein levels of Beclin1 and ATG12‐ATG5 conjugate (markers of the initiation and elongation of autophagic flux) in the RSC of A_2A_R KO mice with TBI were markedly increased (Figure 3D‐F), the levels of LC3‐II (an important marker of autophagosomes) in the A_2A_R KO mice were significantly decreased (Figure 3D,G), and the levels of SQSTM1 (a receptor protein that targets cargo for degradation by autolysosomes) in the A_2A_R KO mice were substantially decreased (Figure 3D,H). Immunohistochemistry revealed similar changes in the levels of LC3 and SQSTM1 in the RSC between WT and A_2A_R KO animals with TBI (Figure S1A‐C). In addition, the TUNEL assay found that the number of apoptotic cells in the injured RSC of WT mice with TBI was more than that in A_2A_R KO mice (Figure S1D,E). The immunofluorescence results indicated that compared with the colocalization in WT mice on the first day after moderate TBI, the colocalization of NeuN and LC3 in the RSC of A_2A_R KO mice with TBI was significantly decreased (Figure 4A,B), and a similar decrease was observed in the colocalization of caspase‐3 and LC3 (Figure 4C,D). Mice were infected with AAV‐RFP‐GFP‐LC3 3 weeks before moderate TBI. Compared to the WT mice, the A_2A_R KO mice showed a significantly decreased percentage of yellow (red + green) puncta, which indicate autophagosomes, while the percentage of red puncta, which indicate autolysosomes, was increased in the RSC on the first day after TBI (Figure 4E,F). We previously reported that autophagic flux in the ipsilateral cortex is normal after mild TBI and is impaired after moderate or severe TBI. A_2A_R KO obviously improves the autophagic flux in the ipsilateral cortex after moderate TBI.^11^ To further explore the relationship between impaired autophagic flux in the RSC after TBI and spatial recognition memory, we used chloroquine (CQ) to block autophagic flux in the RSC immediately after mild TBI. We found that compared to WT mice administered CQ, A_2A_R KO mice administered CQ exhibited a significant increase in the number of entries into and exploration time in the novel arm on the 7th day after TBI (Figure 4G,H).

### 3.4 Lysosomal biogenesis increases in the RSC of A_2A_R KO mice after TBI, and the activation of A_2A_R can reduce lysosomal biogenesis in primary cultured neurons

**FIGURE 5:**
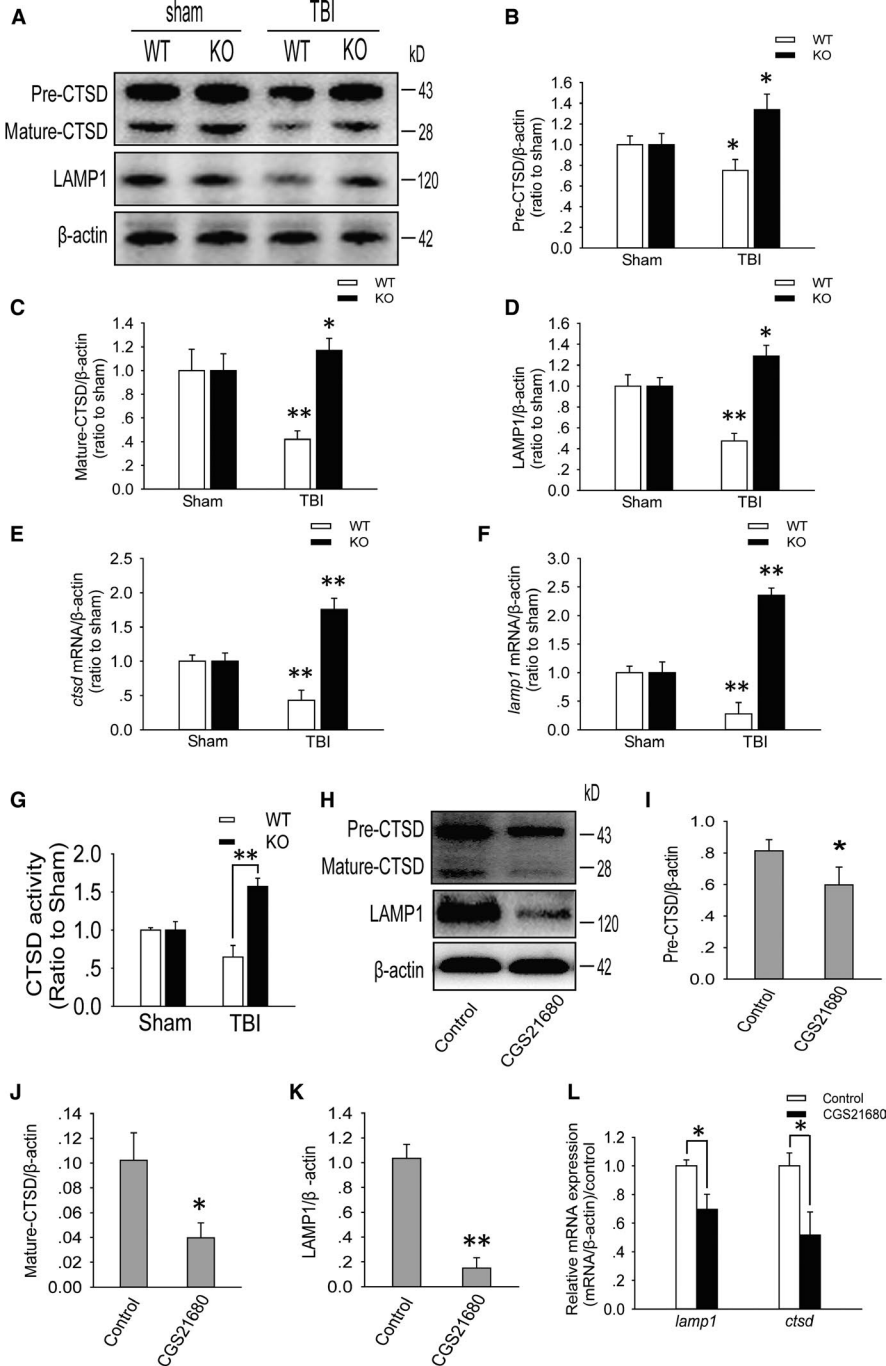
Activated A_2A_R can reduce lysosomal biogenesis in neurons in the retrosplenial cortex after TBI. A, Western blot analysis of LAMP1, pre‐CTSD and mature‐CTSD levels in ipsilateral retrosplenial cortex tissue lysates obtained from mice in the sham, WT + TBI and KO + TBI groups. B‐D, The pre‐CTSD, mature‐CTSD and LAMP1 levels shown in (A) were quantified and normalized to β‐actin. Data shown are presented as the means ± SDs, n = 5, compared to the sham group, *P < 0.05 and **P < 0.01. E and F, Relative mRNA levels (qPCR) of *ctsd* and *lamp1* in the sham and injured mouse ipsilateral retrosplenial cortex. The results were normalized to β‐actin levels. Data are presented as the means ± SDs, n = 3, compared to the sham group, **P < 0.01. G, CTSD enzyme activity, as determined by an in vitro fluorometric assay, in ipsilateral retrosplenial cortex tissue lysates obtained from mice in the sham, WT + TBI and KO + TBI groups. Data are presented as the means ± SDs, n = 6, KO + TBI group vs WT + TBI group, **P < 0.01. H, Western blot analysis of LAMP1, pre‐CTSD and mature‐CTSD levels in primary cultured cortical neurons from the control (DMSO) and CGS21680 groups; DMSO: dimethyl sulfoxide and CGS21680: A_2A_R agonist. I‐K, The pre‐CTSD, mature‐CTSD and LAMP1 levels shown in (H) were quantified and normalized to β‐actin. Data are presented as the means ± SDs, n = 6, *P < 0.05 and **P < 0.01. L, Relative *ctsd* and *lamp1* mRNA levels (qPCR) in primary cultured cortical neurons from the control (DMSO) and CGS21680 groups. The results were normalized to β‐actin levels. Data are presented as the means ± SDs, n = 3, *P < 0.05. KO, knockout; LAMP1, lysosomal‐associated membrane protein 1; TBI, traumatic brain injury; WT, wild‐type

Lysosomal‐associated membrane protein 1 and CTSD are two lysosomal marker proteins. Western blot analysis showed that LAMP1, pre‐CTSD and mature‐CTSD protein levels were significantly decreased in the ipsilateral RSC of WT mice on the first day after moderate TBI compared to those in the sham group of WT mice, while the levels were increased in the ipsilateral RSC of A_2A_R KO mice compared to those in the sham group of A_2A_R KO mice (Figure 5A‐D). Moreover, a similar change was observed in *lamp1* and *ctsd* mRNA levels (Figure 5E,F). At the same time, we found that compared with that in WT mice on day 1 after moderate TBI, CTSD enzyme activity in the RSC of A_2A_R KO mice was significantly increased (Figure 5G). The A_2A_R agonist CGS21680 was added to primary cultured neurons that had undergone oxygen‐glucose deprivation for 2 hours, and we found that LAMP1, pre‐CTSD and mature‐CTSD protein levels were significantly decreased in the treated group compared to those in the vehicle group (Figure 5H‐K). A similar change was observed for *lamp1* and *ctsd* mRNA levels (Figure 5L).

### 3.5 The possible mechanism underlying the regulation of autophagic flux by A_2A_R after TBI

**FIGURE 6:**
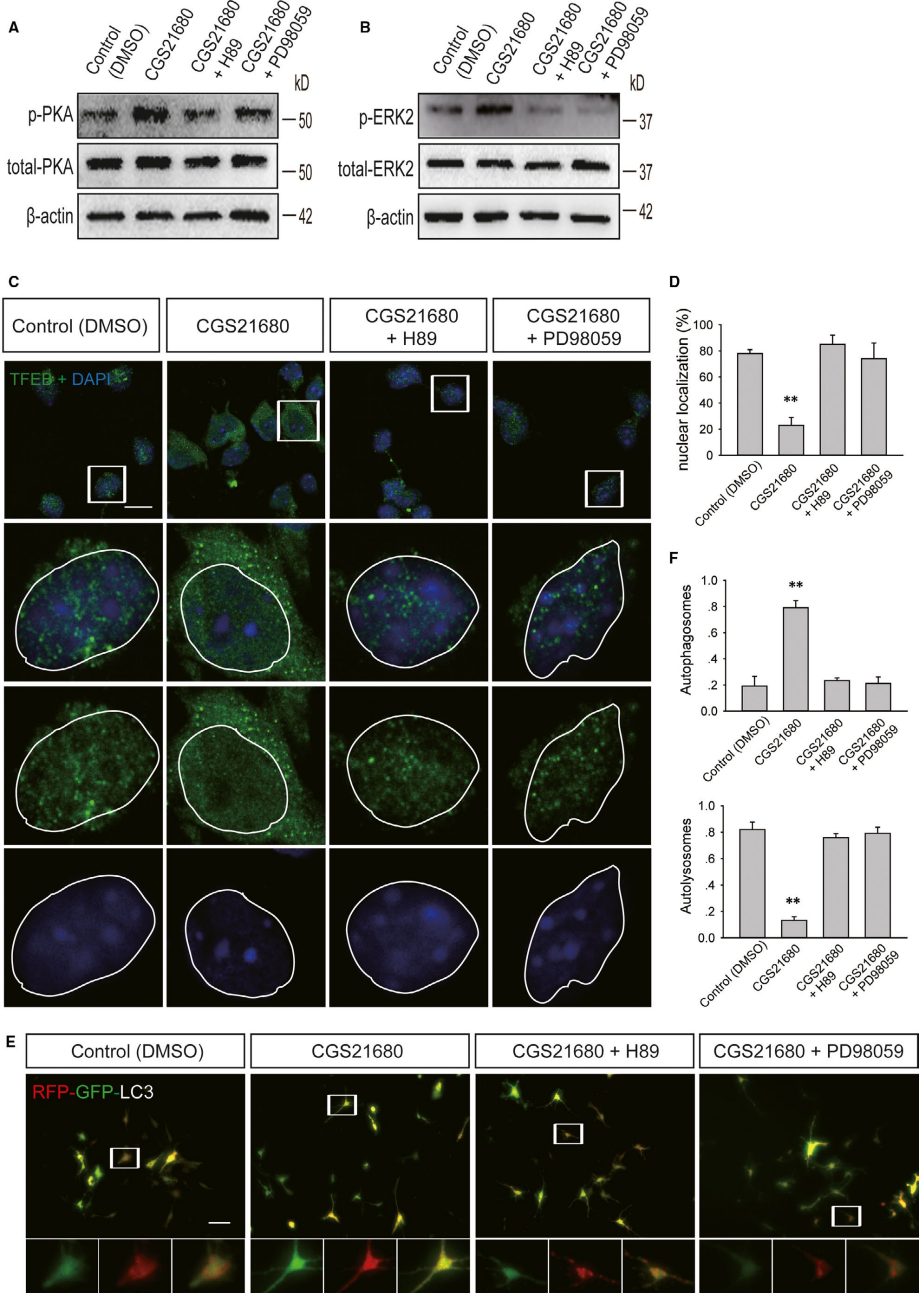
PKA and ERK2 are involved in the regulation of TFEB and autophagic flux after A_2A_R activation. A, Western blot analysis of the levels of phosphorylated PKA and total PKA in primary cultured cortical neurons from the control (DMSO), CGS21680, CGS21680 + H89 and CGS21680 + PD98059 groups; DMSO: dimethyl sulfoxide, CGS21680: A_2A_R agonist, H89: PKA inhibitor and PD98059: ERK inhibitor. B, Western blot analysis of the levels of phosphorylated PKA and total PKA in primary cultured cortical neurons from the control, CGS21680, CGS21680 + H89 and CGS21680 + PD98059 groups. C, Images of primary cultured cortical neurons from the control, CGS21680, CGS21680 + H89 and CGS21680 + PD98059 groups. Neurons were stained using an anti‐TFEB antibody; green fluorescence represents TFEB. Scale bar = 10 μm. D, Quantification of the number of cells with TFEB localized to the nucleus shown in (C). Data are presented as the means ± SDs, n = 3, **P < 0.01 compared to the control group. More than 100 cells were quantified for each group in each experiment. E, Images of primary cultured cortical neurons infected with Ad‐RFP‐GFP‐LC3 from the control, CGS21680, CGS21680 + H89 and CGS21680 + PD98059 groups. Arrows indicate the presence of red puncta. Scale bar = 20 μm. F, Quantification of the percentage of autolysosomes (red puncta/total puncta) and autophagosomes (yellow puncta/total puncta) in the images shown in (E). Data are presented as the means ± SDs, n = 3, compared to the control group, *P < 0.05. More than 100 cells were quantified for each group in each experiment. GFP, green fluorescent protein; RFP, red fluorescent protein; TFEB, transcription factor EB

**FIGURE 7:**
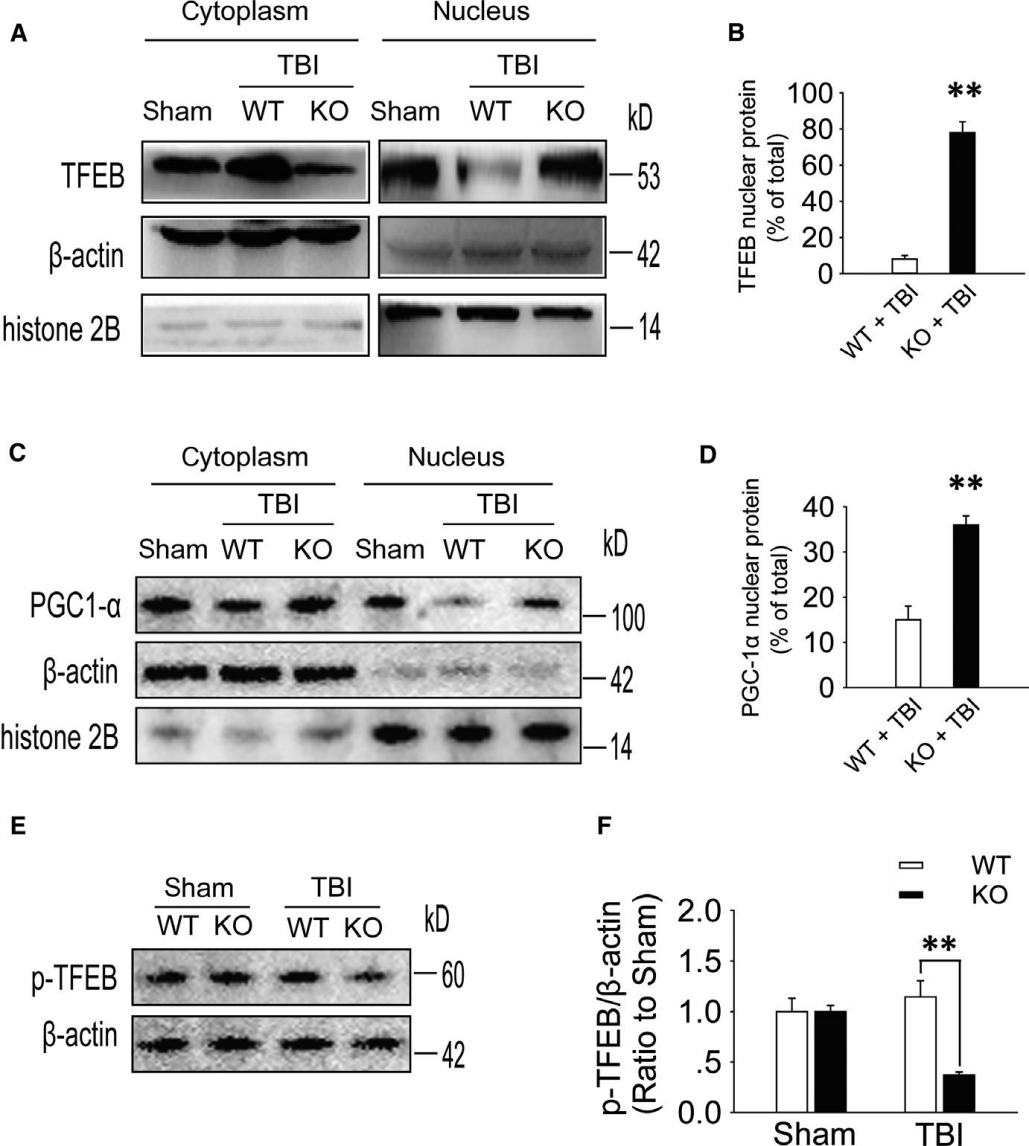
TFEB activation and translocation to the nucleus is reduced by A_2A_R activation after TBI. A, Western blot analysis of TFEB levels in ipsilateral retrosplenial cortex tissue lysates (nucleus and cytoplasm) obtained from mice in the sham, WT + TBI and KO + TBI groups. B, TFEB nuclear content as x % of total (nuclear and cytoplasmic) signal. Histone 2B and β‐actin were used as nuclear and cytoplasmic purity controls, n = 5, KO + TBI group vs WT + TBI group, **P < 0.01. C, Western blot analysis of PGC‐1α levels in ipsilateral retrosplenial cortex tissue lysates (nucleus and cytoplasm) obtained from mice in the sham, WT + TBI and KO + TBI groups. D, PGC‐1α nuclear content as x % of total (nuclear and cytoplasmic) signal. Histone 2B and β‐actin were used as nuclear and cytoplasmic purity controls, n = 5, KO + TBI group vs WT + TBI group, **P < 0.01. E, Western blot analysis of the protein level of p‐TFEB in RSC tissue lysates obtained from sham and TBI mice 1 d after injury. F, The p‐TFEB levels shown in (E) were quantified and normalized to β‐actin. Data are presented as the means ± SDs, n = 6, KO + TBI group vs WT + TBI group, **P < 0.01. KO, knockout; RSC, retrosplenial cortex; TBI, traumatic brain injury; TFEB, transcription factor EB; WT, wild‐type

Drugs were added to primary cultured neurons that had undergone oxygen‐glucose deprivation for 2 hours. Compared with those in the vehicle, CGS21680 + H89 and CGS21680 + PD98059 groups, the protein levels of p‐PKA and p‐ERK2 were increased in the CGS21680 group (Figure 6A,B), while the number of cells with TFEB nuclear localization was significantly decreased (Figure 6C,D). Neurons were infected with Ad‐RFP‐GFP‐LC3 3 days before drugs were added and the cells were subjected to oxygen‐glucose deprivation for 2 hours. Compared with the percentage in the other groups, the percentage of yellow puncta was significantly increased, while the percentage of red puncta was decreased in neurons in the CGS21680 group (Figure 6E,F). Compared to WT mice, A_2A_R KO mice showed an increased level of nuclear TFEB in the RSC on the first day after TBI (Figure 7A,B). Moreover, we also found that compared with WT mice, A_2A_R KO mice showed an increased level of nuclear PGC‐1α in the RSC on day 1 after TBI (Figure 7C,D). At the same time, the level of p‐TFEB in the RSC of A_2A_R KO mice after TBI was lower than that in WT mice (Figure 7E,F).

## 4 DISCUSSION

### 4.1 Decreased function in the RSC after TBI is an important factor in spatial recognition memory impairment

We found that ipsilateral RSC function was significantly decreased and that spatial recognition memory was impaired after moderate TBI. Additionally, we optogenetically inhibited the function of the RSC and found that spatial recognition memory was impaired. The above results indicate that the function of the RSC is closely related to spatial recognition memory. Many studies have found that spatial recognition memory is associated with the hippocampus, septum, basal forebrain and prefrontal cortex.^21^, ^22^, ^23^, ^24^ Furthermore, many researchers have suggested that the RSC functions to integrate spatial information in the above brain regions and that impairment of RSC function is associated with memory impairment in many nervous system diseases.^25^, ^26^, ^27^, ^28^ However, no direct evidence has shown that the RSC participates in the spatial recognition memory loop. Therefore, the results from the present study have, for the first time, confirmed that decreased RSC function may be an important factor in the impairment of spatial recognition memory in mice after TBI. Further research is needed to confirm whether RSC is the most unique brain region for spatial recognition memory.

### 4.2 Inhibition of A_2A_R in the RSC can alleviate spatial recognition memory impairment after TBI

The expression of A_2A_R was significantly increased in the ipsilateral RSC of WT mice after moderate TBI, while ipsilateral RSC function and spatial recognition memory impairment were improved in A_2A_R KO mice, indicating that A_2A_R activation in the ipsilateral RSC may lead to decreased RSC function and impaired spatial recognition memory. To further clarify the relationship between A_2A_R activation in the ipsilateral RSC and the impairment of spatial recognition memory after moderate TBI, we used the A_2A_R antagonist ZM241385 to specifically inhibit A_2A_R activation in the ipsilateral RSC after TBI. We found that spatial recognition memory was significantly improved compared to that in the vehicle group, suggesting that A_2A_R activation in the ipsilateral RSC is associated with spatial recognition memory impairment after moderate TBI. Many studies have found that intervention through other means can improve the cognitive function of mice by reducing adverse events such as neuroinflammation, oxidative stress or brain oedema after TBI.^4^, ^6^, ^7^, ^29^ Moreover, we know that inhibiting A_2A_R has multitarget effects on reducing neuroinflammation, oxidative stress and brain oedema after TBI.^20^, ^30^, ^31^, ^32^ Therefore, the findings of the current study further confirm that the regulation of A_2A_R can improve the cognitive function of mice by reducing the occurrence of adverse events after TBI.

### 4.3 Inhibition of A_2A_R can alleviate spatial recognition memory impairment after TBI and is associated with improved autophagic flux and reduced apoptosis in RSC neurons

The current study found that compared to those in WT mice, the accumulation of autophagosomes in neurons and neuronal apoptosis in the ipsilateral RSC were reduced in A_2A_R KO mice on the first day after moderate TBI. These results indicate that A_2A_R activation in the ipsilateral RSC may be an important cause of autophagosome accumulation in neurons. Furthermore, we found that compared with those in WT mice, the induction of autophagy and autolysosomal function were enhanced in the RSC of A_2A_R KO mice on the first day after moderate TBI. At the same time, we also found that the percentage of yellow puncta representing autophagosomes was significantly decreased, while the percentage of red puncta representing autolysosomes was increased in the RSC neurons of A_2A_R KO mice on the first day after moderate TBI compared to those in WT mice. Both of the above results indicate that the reductions in accumulation of autophagosomes in neurons and neuronal apoptosis are due to improved autophagic flux. Increased apoptosis after spinal cord injury is the main reason for the reduced T1 signal detected by MEMRI;^33^, ^34^, ^35^ that is, apoptosis in the spinal cord is the main cause of reduced spinal cord function. Therefore, in our study, neuronal apoptosis induced by the impairment of autophagic flux by A_2A_R activation may be an important cause of reduced function of the RSC after moderate TBI. In our previous study, we found that autophagic flux in the ipsilateral cortex was normal after mild TBI and was impaired after moderate and severe TBI. A_2A_R KO obviously improves autophagic flux in the ipsilateral cortex after moderate TBI.^11^ Thus, in the current study, we used CQ to block autophagic flux in the ipsilateral RSC of WT and A_2A_R KO mice after mild TBI. We found that spatial recognition memory was obviously impaired in CQ‐treated WT mice compared to that in CQ‐treated A_2A_R KO mice, further indicating that the alleviation of spatial recognition memory impairment through the inhibition of A_2A_R in the ipsilateral RSC after TBI is related to improved autophagic flux in neurons and a reduction in apoptosis. Although the RSC is not the only brain area involved in spatial recognition memory, the above results indicate that the RSC may play a key role in spatial recognition memory after TBI.

### 4.4 Activation of A_2A_R may affect lysosomal biogenesis and impair autophagic flux after TBI through the PKA/ERK2/TFEB pathway

Autophagosomes combine with lysosomes to form autolysosomes under normal conditions and then autolysosomes degrade metabolic waste wrapped inside autophagosomes.^36^, ^37^ We observed that impaired autophagic flux in RSC neurons resulted in the accumulation of autophagosomes. Meanwhile, the protein and mRNA levels of LAMP1 and the lysosomal enzyme CTSD were significantly decreased in WT mice but were significantly increased in A_2A_R KO mice after moderate TBI. Moreover, CTSD enzyme activity in WT mice was also lower than that in A_2A_R KO mice after TBI. These results suggest that the impaired autophagic flux may be related to the inhibition of lysosomal biogenesis by A_2A_R activation and that a decreased number of lysosomes led to decreased autolysosome formation. We also found that A_2A_R activation by CGS21680 significantly reduced lysosomal biogenesis in primary cultured neurons compared to that observed in the vehicle group, which further supports that A_2A_R activation may lead to lysosomal biogenesis disorder and subsequently impair autophagic flux.

Transcription factor EB plays an important role in the expression of autophagy‐ and lysosome‐related genes. TFEB localizes to the cytoplasm when it is phosphorylated, and it can enter the nucleus to participate in autophagy‐ and lysosome‐related gene expression when it is dephosphorylated.^38^, ^39^ In primary cultured neurons that underwent oxygen‐glucose deprivation, the levels of p‐PKA and p‐ERK2 were increased, the nuclear localization of TFEB was decreased, and the percentage of yellow puncta representing autophagosomes was significantly increased, while the percentage of red puncta representing autolysosomes was decreased after A_2A_R activation. These results suggest that A_2A_R activates the PKA/ERK2/TFEB pathway to impair autophagic flux. In animal experiments, we found that TFEB and PGC‐1α were mainly located in the cytoplasm of WT mice after moderate TBI but were mainly located in the nuclei of A_2A_R KO mice. At the same time, we found that the p‐TFEB (Ser142) level in WT mice after TBI was higher than that in A_2A_R KO mice. These results also provide support for the above mechanism. This conclusion is consistent with the findings of Settembre et al, who examined the relationship between TFEB and autophagy and showed that ERK2 can regulate TFEB nuclear localization.

### 4.5 Conclusion

In summary, this study is the first to show that decreased RSC function is an important factor in spatial recognition memory impairment in mice after TBI. While decreased RSC function is related to impaired autophagic flux in neurons and increased neuronal apoptosis via A_2A_R activation, this mechanism may underlie the inhibition of lysosomal biogenesis through the PKA/ERK2/TFEB pathway, leading to autolysosome formation dysfunction. These results provide an experimental basis for elucidating the mechanism of spatial recognition memory impairment after TBI and preventing cognitive dysfunction by improving autophagy function by targeting A_2A_R after TBI.

## CONFLICT OF INTEREST

The authors declare that they have no conflict of interest to disclose.

## AUTHOR'S CONTRIBUTIONS

YGZ and XJZ designed the research, analysed the data and wrote the paper; XJZ, YP, NY and YWX performed the research under the overall co‐ordination of YGZ; PL, YLN, YZ and JFC analysed the data; PL, YLN, YZ, JFC and YGZ supervised the research.

## Supporting information

Fig S1

Fig S2

## Data Availability

The data that support the findings of this study are available from the corresponding author upon reasonable request.
