# Supplementary figures and images for: A2AR inhibition in alleviating spatial recognition memory impairment after TBI is associated with improvement in autophagic flux in RSC
Source: J Cell Mol Med. 2020 May 12;24(12):7000–14. doi: 10.1111/jcmm.15361 (PMC7299719; doi:10.1111/jcmm.15361)

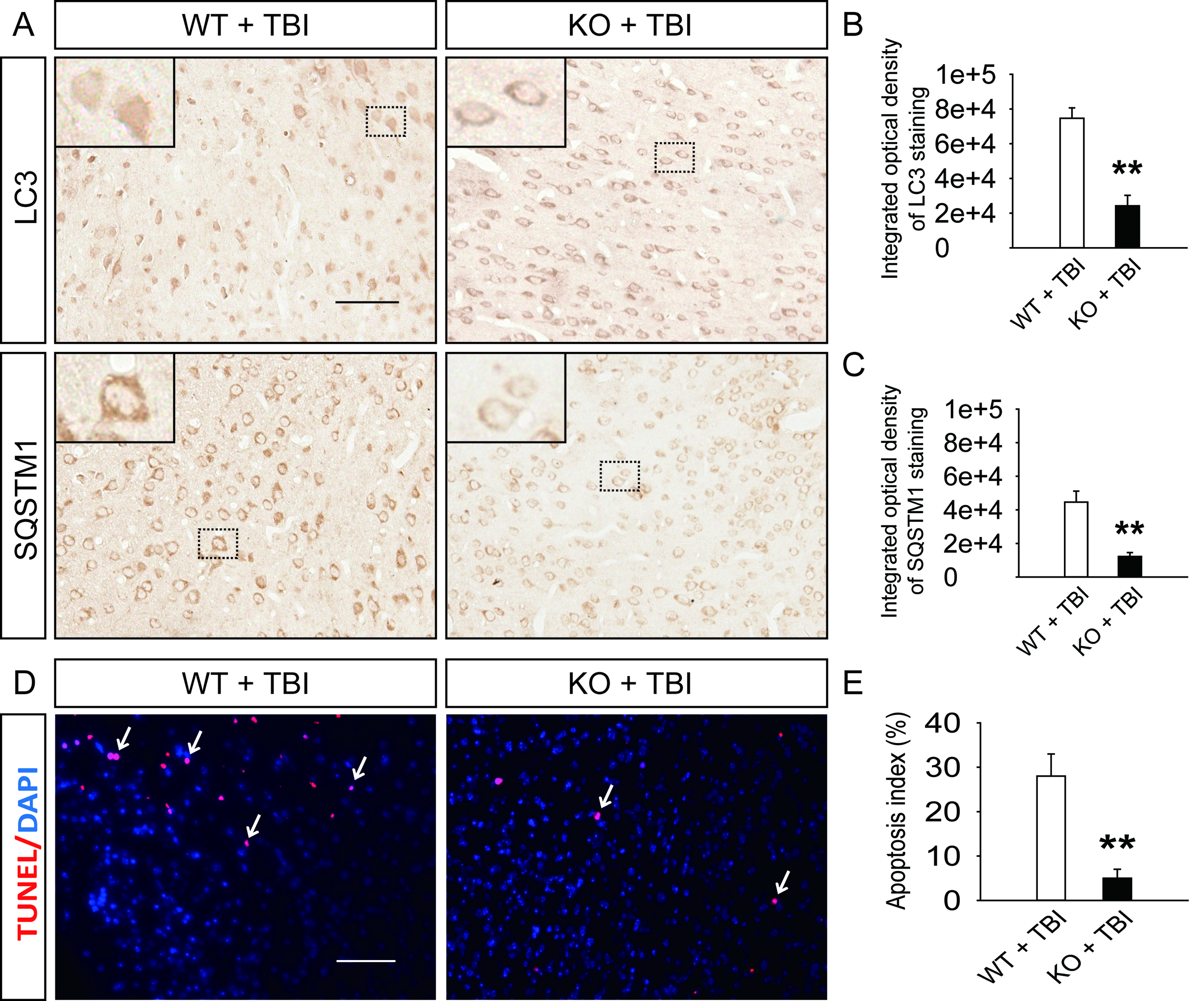

Supplement: Supplementary file 1 — Fig S1 [file JCMM-24-7000-s001.tif]

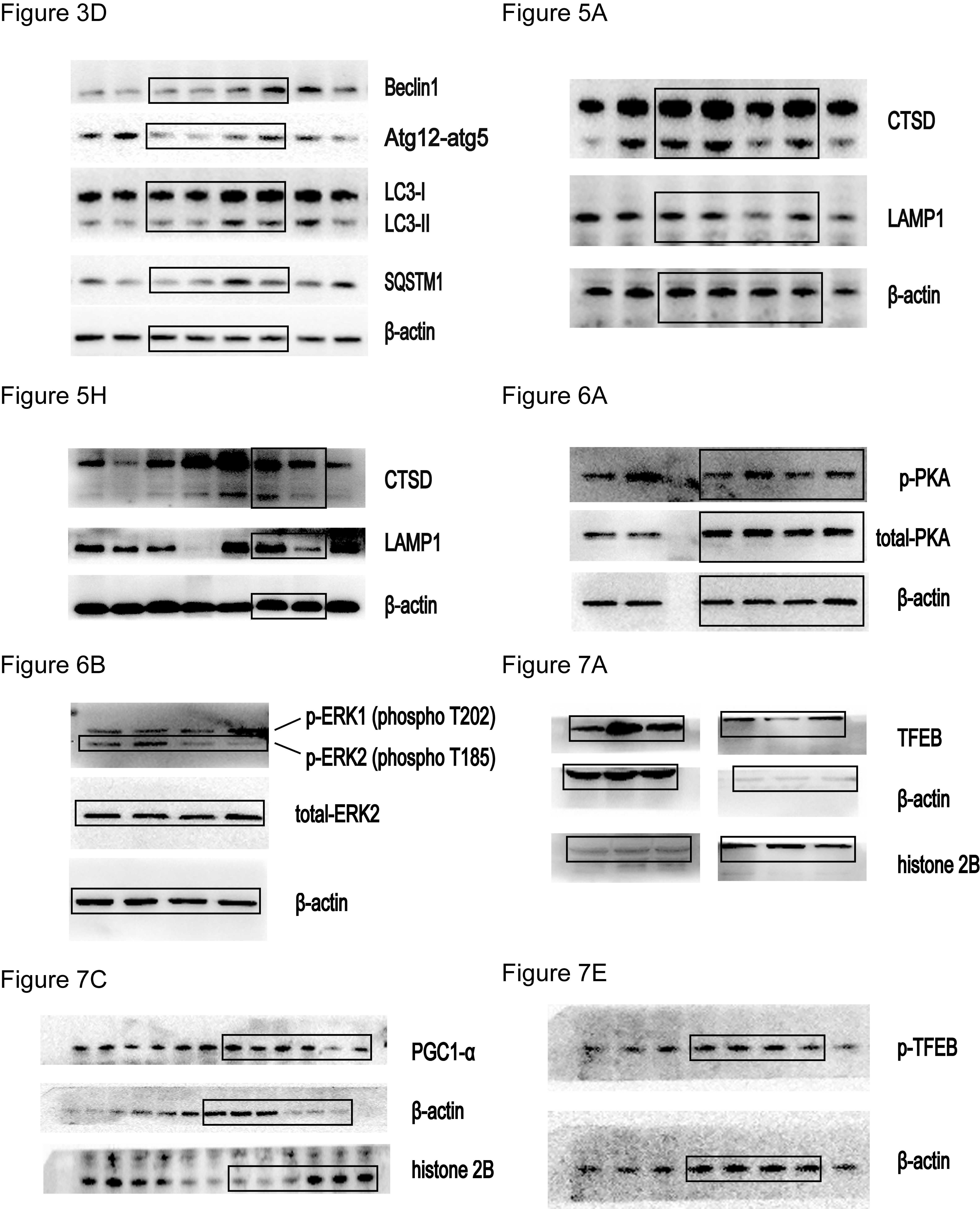

Supplement: Supplementary file 2 — Fig S2 [file JCMM-24-7000-s002.tif]
